# Mobility of A Water Droplet on Liquid Phase of N-Octadecane Coated Hydrophobic Surface

**DOI:** 10.1038/s41598-018-33384-0

**Published:** 2018-10-10

**Authors:** Bekir Sami Yilbas, Haider Ali, Abdullah Al-Sharafi, Nasser Al-Aqeeli, Numan Abu-Dheir, Kahraman Demir

**Affiliations:** 1 Mechanical Engineering Department and Centre of Excellence in Renewable Energy, King Fahd University of Petroleum & Minerals, Dhahran, Saudi Arabia; 2 Center of Research Excellence in Renewable Energy (CoRE-RE), King Fahd University of Petroleum and Minerals (KFUPM), Dhahran, 31261 Saudi Arabia

## Abstract

A water droplet behavior on the liquid n-octadecane film is investigated. The coating of hydrophobic surface by N-octadecane film provides exchange of wetting state on the surface. The polycarbonate surface is crystallized and the functionalized silica particles are placed on the resulting surface prior to thin film coating of n-octadecane. A high-speed camera is used to monitor dynamic characteristics of the droplet on the inclined film. The findings reveal that deposition of thin n-octadecane film on hydrophobic surface results in reversibly exchange of the wetting state at the surface, which remains hydrophobic when n-octadecane film is in solid phase while it becomes hydrophilic when n-octadecane film liquefies. Droplet transition velocity predicted agrees well with the experimental data. Sliding mode of the water droplet governs droplet transition on the liquid surface. Droplet pinning force, due to interfacial tension, dominates over the other retention forces including drag and shear.

## Introduction

Droplet mobility on surfaces plays a key role in several applications including water-oil separation^1^, anti-corrosion^2^ anti-icing^3^, drag reduction^4^, self-cleaning^5^, and similar. Surface hydrophobicity with low contact angle hysteresis remains critical for droplet mobility on the surfaces. Droplet mobility is suppressed significantly by high contact angle hysteresis, which in turn creates pinning effect of the droplet on the surface. Generating lotus effect on the surface towards minimizing the droplet pinning requires surface texturing with hierarchically distributed micro/nano size pillars. This necessitates incorporating multiple processing using chemical agents or precision machining. Some of the surface texturing processes are expensive and involves with the harsh conditions. However, the droplet mobility remains high on the liquid impregnated surfaces, which does not require efforts for surface texturing in harsh environments. The liquid impregnation of surfaces requires positive spreading coefficient (*S*) to form a liquid film on the substrate surface^6^. This limits the selection of the liquids for impregnation process. In addition, the surface texture characteristics, such as surface energy and roughness parameter (the ratio of the area of texture to the solid surface projected area) influence the spreading coefficient of the liquid on the surface^7^. On the other and, varying the wetting state from hydrophobic to hydrophilic and vice versa of the surface is favorable for some applications^8,9^. This becomes particularly interesting if the change of the wetting state takes place reversibly. One of the methods to achieve reversible wetting state is to deposit a thin layer of phase change material on to the hydrophobic surface^10^. This arrangement provides the surface wetting state change in such a way that the surface becomes hydrophilic when the thin liquid film is in the liquid phase and it remains hydrophilic when the film is in the solid phase^10^. However, the droplet behavior on the liquid film formed from the phase change material is important in terms of practical applications of reversible exchanging of the surface wetting state. Consequently, investigation of the droplet behavior on the liquid film of the phase change material becomes necessary.

The droplet mobility on the liquid surfaces were investigated previously. Droplet spreading on a surface exhibiting solid-liquid interfacial pre-melting was studied by Yang and Laird^11^. They demonstrated that when pre-melting was suppressed, the equilibrium contact angle was observed to be significantly larger than when pre-melting was present even at same temperature ranges; hence, the presence of pre-melting layer had a significant effect on the properties of the solid-liquid interface, but little effect on the mechanism of spreading. In addition, the structure of the droplet contact line in the presence of the pre-melting layer could be described by two contact angles (instead of the usual one) because of the presence of additional pre-melting layer. Water droplet motion on the asymmetrically patterned surfaces was examined by Chamakos *et al*.^12^. They showed that the unbalanced capillary force, developed at the contact line, was the key factor for achieving a preferable liquid motion direction. A sliding droplet on an asymmetrically structured surface exhibited different migration velocities depending on the direction of the structures with respect to the motion, only when the capillary forces were predominant against the effect of inertia. The anisotropic wetting properties, due to structure asymmetry, could possibly be exploited in order to passively transfer a droplet by vibrating the substrate, either vertically or horizontally. A single droplet impact onto a liquid film with horizontal velocity was examined by Liu *et al*.^13^. They indicated that the behavior of resultant splash structure after droplet impact was greatly affected by Bond (Bo) and Weber (We) numbers. The dynamics of a spherical droplet, which is partially immersed in a semi-infinite phase of a micropolar fluid, was studied by Saad^14^. He presented the analytical solution for the stream functions outside and inside of the droplet during its movement. In addition, he evaluated the drag force acting on the part of fluid sphere immersed in the micro-polar fluid. The electro-kinetic motion of a spherical polystyrene particle at a liquid-fluid interface was investigated by Zhang *et al*.^15^. They showed that magnitude of the particle velocity increased linearly with the increasing applied electric field. For particles of same sizes, the electro-kinetic velocity at the liquid-fluid interface was larger than particle electrophoretic velocity in the liquid bulk. The directional motion of a single droplet on cactus spines was examined by Guo and Tang^16^. The findings revealed that both Newtonian droplet and shear-thinning non-Newtonian droplet moved directionally from the tip to the base owing to the decreased Gibbs free energy along the spine. The non-Newtonian droplet moved slower because of the increased viscosity loss due to shear-thinning. A study on the behavior of liquid droplets on gradient surfaces was carried out by Zhu *et al*.^17^. They demonstrated that the height and density of the silane molecule groups determined the surface energy distribution on the surface. The liquid droplets were self-propelled to move horizontally or uphill from hydrophobic zone to hydrophilic zone on the gradient surface. The droplet experienced an accelerating and creeping decelerating stages on the surface; the velocity and the displacement as well as the creeping frequency were proportional to the droplet size. The axial motion of the small droplets deposited on smooth and rough fiber surfaces was investigated by Gac and Gradon^18^. They derived the expression for the droplet velocity and demonstrated that the distinguished patterns of the interaction depending on the structure of the fiber roughness and the fiber and the droplet dimensions. The electrohydrodynamic behavior of water droplets on a horizontal superhydrophobic surface was studied by Li *et al*.^19^. The findings revealed that the micro/nano hierarchical surface structure and chemical components with low surface free energy of the superhydrophobic surface jointly contributed to the reduction of skin friction drag and subsequently made it possible for the motion of the water droplet driven by applied electric field. The propelled motion of the water droplets could also sweep contaminations along its moving trace, which provided superhydrophobic surface a promising anti-pollution prospect in power systems.

Although droplet mobility on oil impregnated surface was studied previously^7^, the main focus was to introduce a scaling model incorporating the viscosity of lubricant and elucidating the dependence of drag reduction on the ratio of viscosity of working fluid to that of the lubricant. In addition, the water droplet mobility on a hydrophobic surface under a thermal radiative heating was investigated^20^; however, the influence of gravitational potential energy on the droplet motion on the inclined impregnated surface was left for the future study. The exchange of the surface wetting state is demonstrated to be possible when the surface is coated by a thin layer of a phase change material^10,21^; however, the droplet mobility on the liquid phase of the thin film of the phase change material needs to be examined further. This becomes essential to assess water droplet behavior on the reversibly exchanging wetting state surface, if the surface remains in a liquid phase. Consequently, in the present study, water droplet behavior on the liquid film formed from the phase of phase material is investigated in relation to wetting state exchange of the surface. Polycarbonate surface is crystallized applying the acetone solution towards creating the hydrophobic surface texture with micro/nano size texture characteristics. To reduce the contact angle hysteresis and increase the droplet contact angle on the surface, the nano-size silica particles are deposited on the textured surface. A thin layer of phase change material (n-octadecane) is formed on the particles deposited surface towards achieving the wetting state exchange of the surface. The water droplet dynamics are examined for the inclined n-octadecane film formed surface.

## Experimental

Polycarbonate wafers (30 × 80 mm×3 mm, width, length and thickness) were prepared for acetone acetone induced crystallization of surface in accordance with the early studies^21,22^. The textured surface via crystallization was coated by the nano-size silica particles. This arrangement provided increased contact angle and reduced contact angle hysteresis. The nano-size silica particles were formed incorporating the chemical process^21^. In this case, the synthesizing process involved with the mixture of isobuthytrimethoxysilane (OTES), t etraethyl orthosilicate (TEOS), 3-aminopropyltrimethoxysilane (AMPTS), ethanol, and ammonium hydroxide. The final mixture was placed on mechanical shaker for 18 hours at room temperature. The solution was centrifuged first and later washed with ethanol for the removal of chemical reactants. The solvent casting was introduced for the deposition of the particles on the textured polycarbonate surfaces. The deposited surface was vacuum dried to ensure the solvents was evaporated and removed from the surface. Later, the characterization study was carried out to analyze the surface; in which case, the surface resulted in the droplet contact angle of 158° wand the contact angle hysteresis of 2°.

The morphology of the textured and coated surface was examined incorporating the focused ion beam (FIB) field emission scanning electron microscope (FESSEM). The texture characteristics were assessed using the atomic force microscope (AFM). The AFM probe was silicon nitride (*r* = 20–60 nm) and the probe force constant, *k*, was of 0.12 N/m.

A liquid film of n-octadecane phase change material was formed on the surface of the silica particles coated polycarbonate wafer. The technique of the dip coating was adopted using the coating unit (Chemat Scientific KW 4AH, Chemat Technology Inc., USA). During the coating process, n-octadecane was kept in the liquid form while keeping the temperature constant at 304 K. The thickness of n-octadecane film was measured incorporating the ellipsometer (Model: M-2000 Manufacturer: J.A. Woolam Co., USA). The film thickness measurement was basing on the polarization change, which could be defined through the values of: i) amplitude ratio (Ψ), and ii) phase difference (Δ), i.e. tanΨ*e*^*i*Δ^ = *R*_*p*_/*R*_*s*_, here *R*_*p*_ and *R*_*s*_ are the *p*- and *s*- polarized light reflection coefficients (Fresnel), respectively. The ellipsometer produced the data for the amplitude ratio and the phase difference at each wavelength of the optical radiation while generating the resulting spectrum data. The thickness of the n-octadecane film was measured to be 40 μm ± 20 nm.

A goniometer (Kyowa, model DM 501) was incorporated to carry out the droplet contact angle measurements. Desalinated water was utilized in the tests and the volume of the droplet was adjusted via an automatic dispensing system. The droplet images were recorded one second after droplet deposition on the surface.

## Results and Discussion

Water droplet dynamics on the inclined hydrophilic thin liquid film of phase change material is examined. A thin film of n-octadecane coating on the surface of the crystalized and silica particles deposited wafer resulted in exchange of the surface wetting state. Hence, surface remains hydrophobic for the solid phase of n-octadecane, and it becomes hydrophilic once the solid phase melts and forms a thin liquid film.

### Morphology of n-octadecane and Nano-size Silica Particles Coated Surface

**Figure 1:**
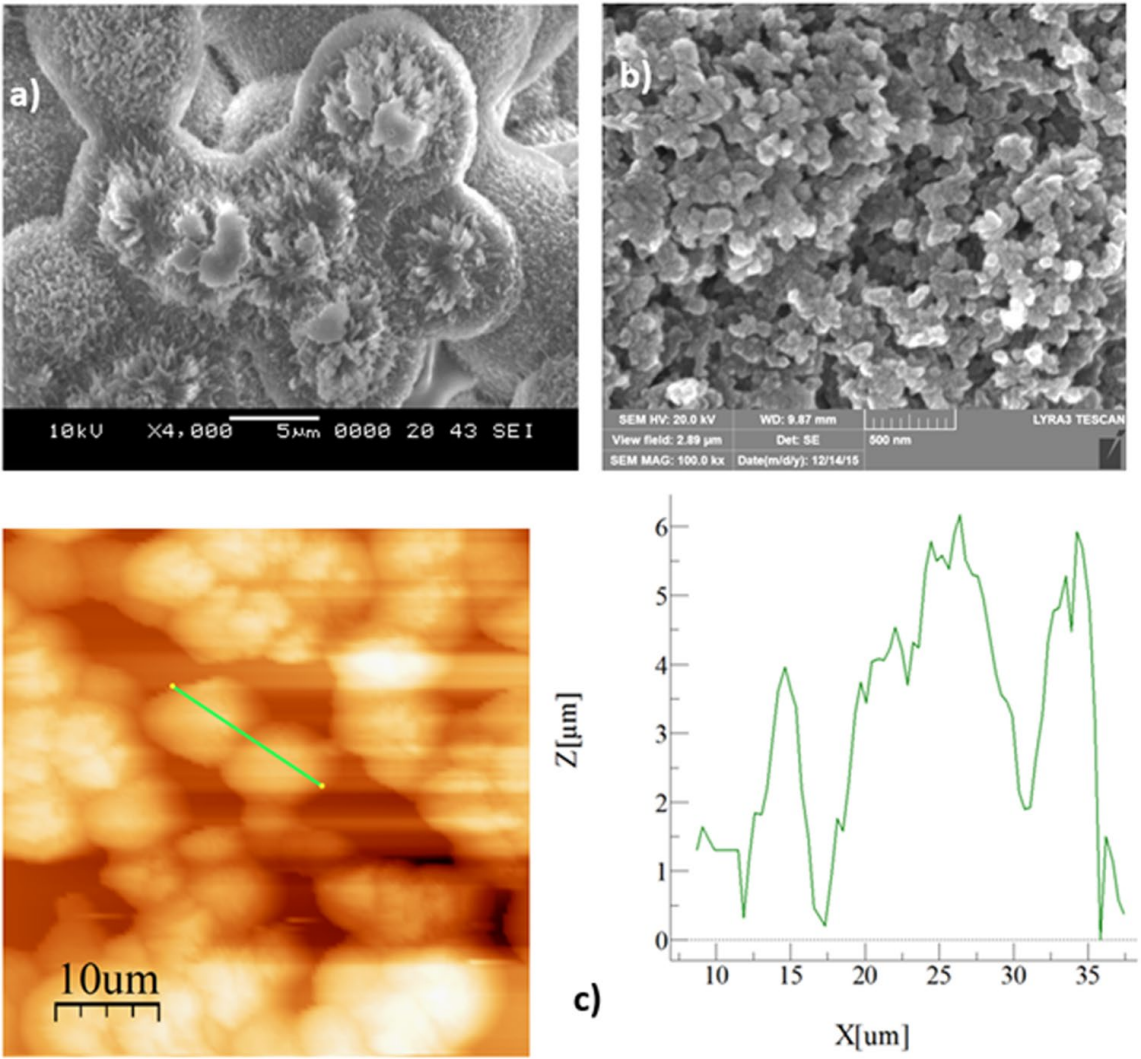
SEM micrographs of crystallized and functionalized particles deposited polycarbonate surface and AFM line scan on the surface: (**a**) SEM micrograph of crystallized surface, (**b**) SEM micrograph of functionalized silica particles deposited surface, and (**c**) AFM line scan of crystallized and functionalized silica particles deposited polycarbonate surface.

The crystallization process results in hierarchically distributed surface texture with micro/nano size fibrils and spherules (Fig. 1a). The spherules and fibrils vary by size resulting in hydrophobic characteristics at the surface. The crystallized surface has the droplet contact of 130° and the contact angle hysteresis of 36°; hence, droplet mobility is suppressed on the surface due to the large hysteresis despite hydrophobic wetting state on the surface. In order to overcome this shortcoming, the crystallized surface was coated with nano-size silica particles (Fig. 1b). Deposited silica particles appear as porous like structures and these are attributed to the silica particles agglomeration and the presence of voids in the deposited layer. Since orthosilicate (TEOS) is used during the silica particles processing, the roughness of the particle surface can be modified through the functionalization process. In this case, the size of the functionalized cells enlarges in the porous regions^23^, which is associated with the monomer condensing units. Hence, their size increases quicker than the nucleation rate^24^. Consequently, this process increases the agglomeration of the particles on the surface. The size of the particles is 30 nm in average and they extend on the spherules surface. The water droplet contact angle measurements are carried out in line with the previous study^25^. The functionalized silica particles create a Lotus effect on the deposited surface and modify the surface wetting state. The contact angle of the droplet increases to 158° while the hysteresis is in the order of 2°. The texture parameter on the surface is evaluated in terms of the surface roughness parameter (*r*), which is the ratio of the textured surface area to its projected area and it is estimated as *r* = 0.88, and average surface roughness is in the order of 3.4 μm, which can be observed from atomic force microscope line scan (Fig. 1c).

**Figure 2:**
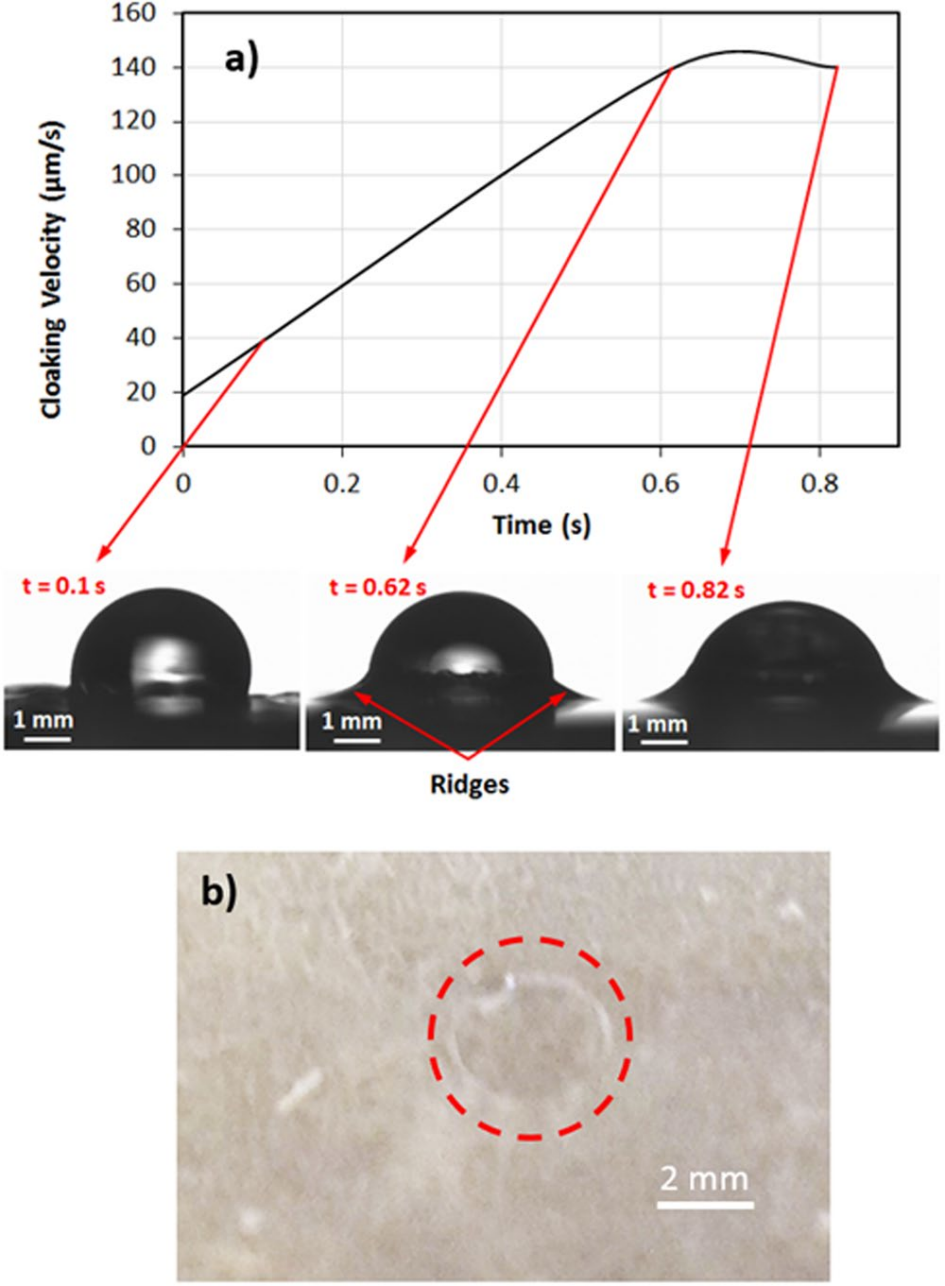
Sessile images of water droplet during cloaking by the liquid phase of n-octadecane and residue of evaporated droplet on the solidified n-octadecane: (**a**) n-octadecane cloaking during different periods and cloaking velocity, and (**b**) water droplet residues on solidified n-octadecane after complete evaporation of water droplet from the surface.

The transition of wetting states is associated with the spreading of the droplet, which depends on the interfacial area and the interfacial surface energies of the droplet fluid on the surface, in air, and free energy of the film surface^26,27^. The roughness parameter is related to the interfacial area where the droplet is located. In the case of the droplet spreading on the liquid film, spreading of droplet and consequently the rate of its spreading are affected by the thickness of the primary film, which, in turn, depends on the distance of the edges from the droplet^27^. In addition, the transition of the wetting state should satisfy the conditions for the transition of Wenzel to Cassie and Baxter states or vice-versa. Depending on the surface energy, the surface texture, and impact of the droplet at the surface, the contact mode changes from Cassie-Baxter state to Wenzel state^28^. Two states can co-exist on a nano-textured surfaces^29^; however, when a droplet fluid-air interface can remain pinned at the pillars tops, transition to the Wenzel state becomes possible. To generate the reversible exchanging of the wetting state at the surface, the textured and silica particles covered surface is coated with the n-octadecane film via adopting the deep coating technique. The phase change temperature of n-octadecane is low such that the solidus temperature is 300.15 K and the liquidus temperature is 303.15 K^30^. Consequently, a small change of film temperature can cause the melting or resolidification of the film on the surface. On the other hand, the rate of liquid film spreading on the particles deposited surface remains critical to: (i) cover and encapsulate the entire surface, and (ii) form a continuous layer of thin film at the coated surface. The liquid film impregnation of the surface is related to the interfacial energy between the liquid film and the surface texture parameter^31^. The liquid film impregnation on the surface can be related to $({\gamma }_{sa}-{\gamma }_{ls})/{\gamma }_{la}=f((1-\phi )/(r-\phi ))$, where *γ*_*sa*_ is the surface energy of textured surface in air, *γ*_*ls*_ is the solid-liquid interfacial energy, and *γ*_*la*_ is the surface energy of the liquid film in air. Here, *φ* is the fraction of the projected area that is not filled by the micro/nano pillars and *r* is the surface roughness parameter. However, introducing the Young’s equation, cos*θ*_*w*_ = *r*(*γ*_*sa*_ − *γ*_*ls*_)/*γ*_*la*_ into the equation that associates with the liquid film impregnation, the Hemi-Wicking criterion for the liquid film should be satisfied^7,8^. Hence, the mathematical arrangements yields the relation cos*θ*_*os*(*a*)_ = (*γ*_*sa*_ − *γ*_*ls*_)/(*γ*_*sa*_ − *γ*_*ls*_), here *γ*_*sa*_ is the interfacial energy between textured surface and air, *γ*_*ls*_ is the interfacial energy between liquid film and the textured surface, and *γ*_*la*_ is the interfacial energy between liquid film and air. The state satisfying the liquid film for impregnating and encapsulating of the textured surface is *θ*_*os*(*a*)_ < *cos*^−1^(1/*r*). In addition, the spreading rate yields *S*_*ls*_ ≡ −*γ*_*ls*_(*r* − 1/*r*); in which case the spreading rate remains *S*_*ls*(*a*)_ ≥ 0^7,8^. Here, *S*_*ls*(*a*)_ is the spreading rate of liquid film on the textured surface in air. The surface energy of the liquid n-octadene is 21.6 mN/m^32^; hence, the spreading rate corresponding to the liquid n-octadecane on the textured surface is in the order of *S*_*ls*(*a*)_ = 2.95 mN/m, i.e. it is larger than zero. The positive spreading rate on the textured surface reveals that the liquid n-octadecane develops a thin layer of film while totally encapsulating the textured surface. However, the flake like micro-size regions are formed on the textured surface once the liquid film solidifies. This can be seen from Fig. 2 in which SEM micrographs are shown for the re-solidified film. The flakes do not form a continuous solid film on the surface rather a patchy type film with irregular unconnected regions in the film. In some regions at the surface the flakes are scattered and the silica particles appear from these sites on the free surface. The area covered by exposed silica particles is about 20% of the total area of the surface. The exposed silica particles from the surface demonstrates the hydrophobic characteristics. In this case, n-octadecane re-solidified surface remains hydrophobic because of the presence of the islands of exposed silica particles despite the fact that the high surface energy of the solid n-octadecane^33^.

In order to assess the wetting state of the solid film on the surface, several tests are conducted to measure the droplet contact angle on the thick layer of n-octadecane solid surface. Hence, 1 mm thick continuous solid film of n-octadecane is developed on the substrate surface and it is used to measure the contact angle on the solid film. The contact angle is measured as 82° and the corresponding hysteresis is about 20°. Moreover, the contact angle is also measured on the re-solidified thin film, which is deposited on the textured surface. The findings demonstrate that the contact angle remains about 140° and the corresponding hysteresis is 8°. Consequently, coating the textured surface with a thin liquid film of n-octadecane results in hydrophobic surface characteristic. However, the wetting state switches from hydrophilic to hydrophobic upon re-solidification of the film on the textured surface. This situation is tested several times and the surface wetting state changes reversibly from hydrophilic to hydrophobic or vice versa depending on the liquid or solid phases of the film.

### Droplet Dynamics on N-octadecane Liquid Film

**Figure 3:**
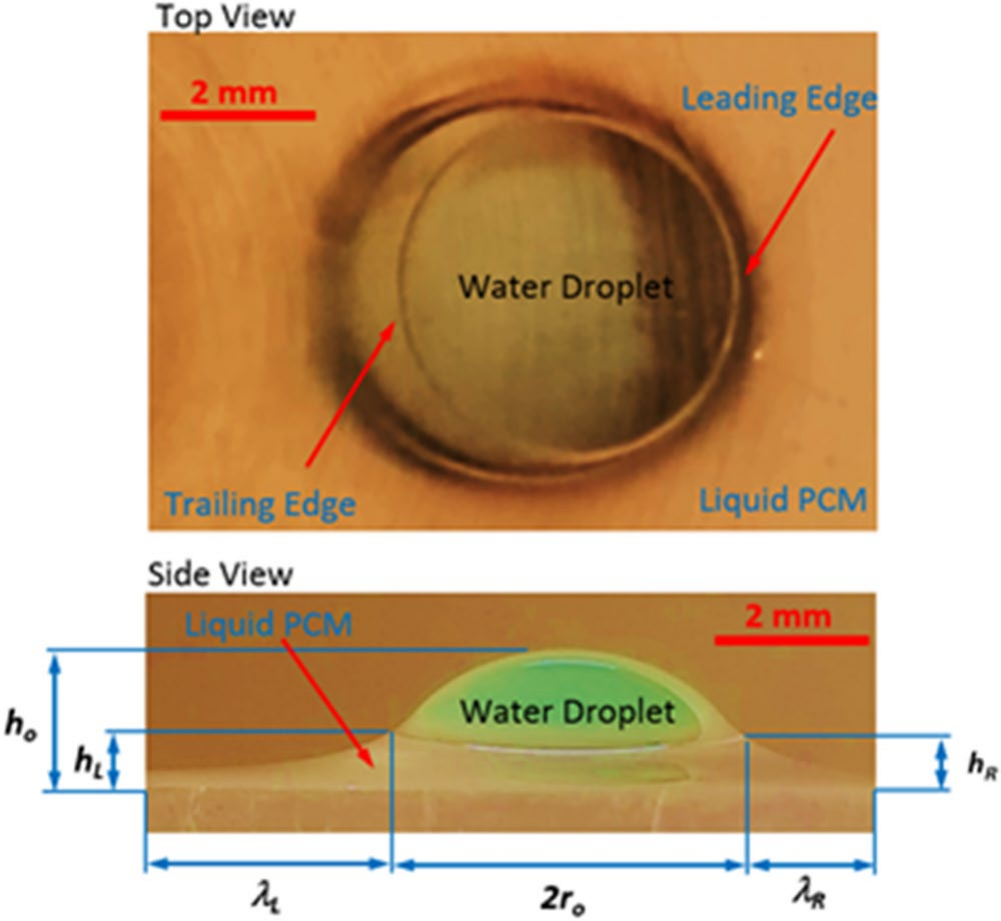
Optical images of water droplet on surface during transition on inclined thin film of liquid n-octadecane. Length scale characteristics of droplet geometry are also shown.

**Figure 4:**
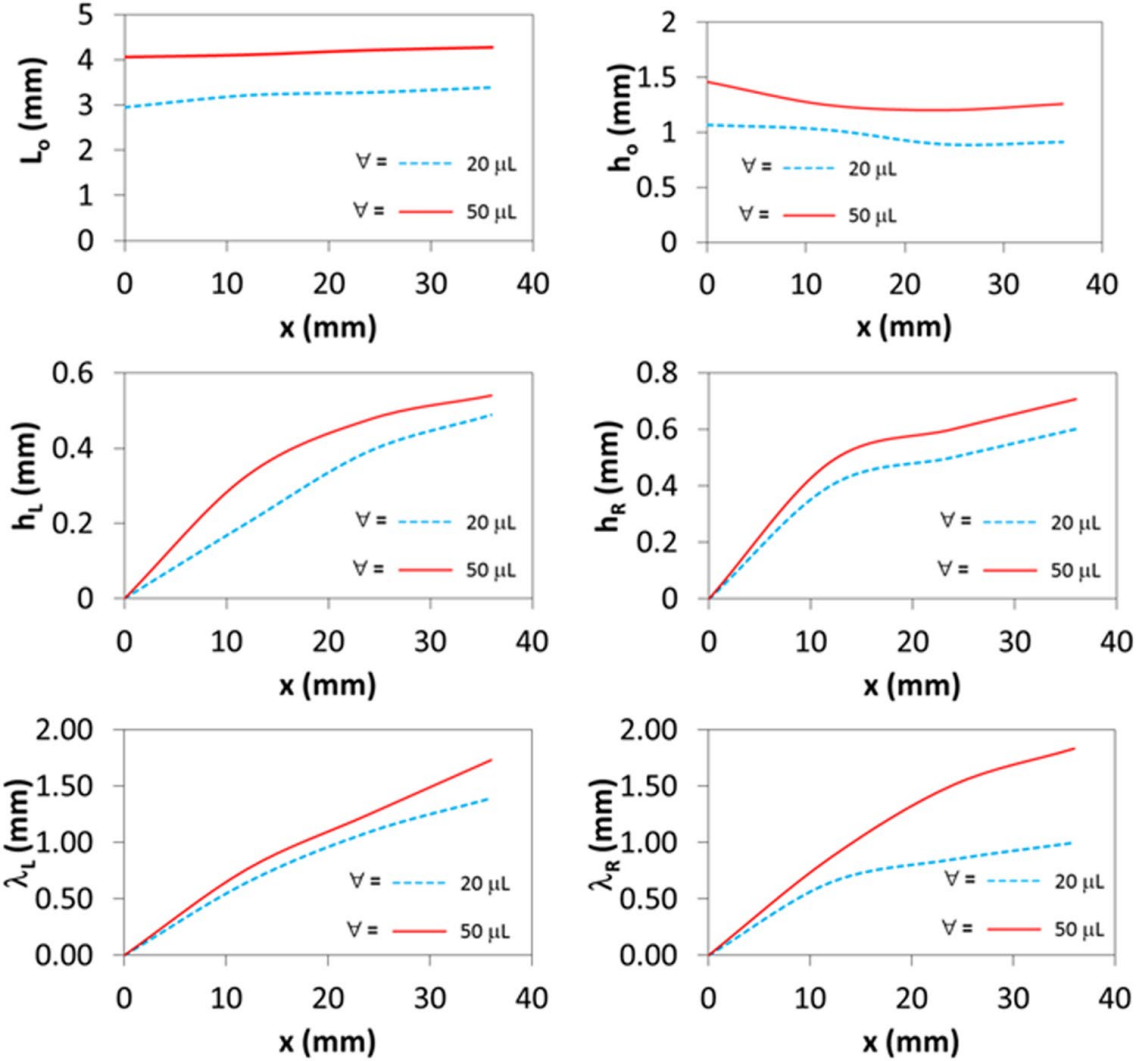
Length scale characteristics of droplet geometry during transition on inclined thin film of liquid n-octadecane.

The water droplet undergoes cloaking on the liquid film surface (Fig. 2a). To determine the cloaking and the height of the ridges developed around the droplet rim in the film, several experiments are carried out. The thickness of the thin liquid film is maintained at 40 μm on the textured surface. Later, temperature of the film is reduced below the solidus temperature (<300.15 K) to solidify the solid the liquid film. Later, the water droplet is formed onto the solid film while the specimen temperature is increased rapidly to re-liquefy the solid film. The solid film on the textured surface gives rise to the hydrophobic characteristic and, upon melting, the surface becomes hydrophilic state. The behavior of the droplet prior and after the rapid heating is recorded incorporating the high-speed camera. Since the ridge is developed around the droplet in the liquid film (Fig. 2a), it resembles the positive spreading rate (*S*_*lw*_ > 0); in which case, the interfacial energy between the droplet and the liquid film (*γ*_*lw*_.) should be smaller than 30.4 mN/m. The velocity of cloaking is evaluated incorporating the high-speed camera data, which is shown in Fig. 2a. In this case, the cloaking rate of liquid film reaches its peak in the early period (∼0.62 s, Fig. 2a). Therefore, the partially encapsulation of the water droplet occurs by the liquid film. For the late stage, temperature of the sample is reduced and the water droplet and the sample are left at temperature below the solidus temperature of n-octadecane for sometimes until the water droplet is completely evaporated from the sample surface. Figure 3b depicts optical image of the solidified film on the textured sface after the droplet evaporation. The residue of ridges formed by the liquid film is evident from Fig. 2b and the liquid film does not encapsulate completely the droplet, which can be associated with the heat transfer from the layer of the ridge surface to the air environment. In this case, temperature of the liquid film in the ridge reduces and it may reach the solidus temperature of the n-octadecane while terminating the cloaking after the ridge formation. Hence, transferring of heat from the liquid film to its air environment reduces temperature in the film during the cloaking process and the film solidifies before encapsulating the entire surface of the water droplet. The ridge size in terms of height and width around the water droplet are measured at various locations on the surface. In addition, the water droplet height and wetting length of the droplet around the ridges are obtained from the high-speed camera data. Figure 3 shows the geometric arrangements of the droplet and ridge while Fig. 4 shows the sizes of the ridge and droplet determined from the high-speed camera for two droplet volumes (20 μL and 50 μL). It should be noted that in order to observe the contact line around the ridges and the droplet height, the droplet liquid is colored in blue prior to its deposition on the surface. Since the image of the droplet on the mid-way of the inclined surface is shown in Fig. 4, the geometric feature of the ridge in the droplet front, in the sliding direction, is slightly different than that of the droplet behind.

The fluid resistance and pinning force are responsible for the formation of slightly large ridge height in front of the droplet. In this case, stagnation region around the droplet front causes hydrostatic pressure increasing in the ridge while resulting in fluid height elevation in this region. This is more pronounced with increasing droplet volume. The ridge height is associated with the vertical force balance of the interfacial tensions at inflection point^33^. Consequently, the vertical force balance among the gravitational, buoyancy, and surface tension forces alters with changing the droplet volume. The ridge heights difference, due to in front and behind the droplet, remains almost uniform with increasing distance along the inclined surface. The small difference along the short distance from the initiation of the droplet movement is because of the droplet acceleration on the inclined liquid film surface. However, once the initial acceleration is completed, the droplet movement becomes almost steady on the inclined liquid n-octadecane surface. The length of the disturbance on the liquid film surface, due to ridge formation, remains larger in the region behind the droplet than that of the droplet front, which is similar to the wake flow behind a moving object. The contact length along the front and back ridges increases slightly with increasing distance traveled by the droplet on the inclined liquid surface. The droplet height (*h*), which is measured from the reference liquid surface to the droplet tip point, reduces slightly along the hydrophobic surface. This indicates that droplet immerses slightly into the liquid layer of n-octadecane during its motion on the liquid surface.

The surface tension force of the water droplet on the liquid surface, prior and after the cloaking, can be formulated through the analogy of the three-phase contact line around the ridges. In this case, the surface tension force becomes:

1 $${F}_{\gamma }=2\pi r{\gamma }_{w-pcm}$$

where *γ*_*w*−*pcm*_ is the interfacial surface energy between liquid film and water, *R* is the radius of the water cap at the meniscus (rim) of the ridge. The maximum value of *r* reaches to *r*_*o*_, which is associated with the initial cap radius (prior to droplet immersion). After assuming that the initial shape of the droplet on the liquid film resembles the spherical cap with height *h*_*o*_ and diameter *r*_*o*_, then the radius of the three-phase contact line can be written as $r=\sqrt{(2{r}_{o}-h)h}$, where *h* is the spherical cap height, *r*_*o*_ is the initial radius of the spherical cap and it is related to initial height of the cap (*h*_*o*_) and spherical water droplet radius, ${r}_{d}=\frac{4}{9}{h}_{o}(\frac{{r}_{o}^{3}}{{h}_{o}^{3}}+1)$, here *r*_*d*_ is the water droplet radius with spherical geometric feature prior to dispensing onto a liquid phase of n-octadecane (prior to forming a shape of a spherical droplet cap on the surface). The details of formulation of the spherical cap are given in Supplementary Material S1. The interfacial surface tension between the liquid n-octadecane and water can be obtained from the Young’s equation^34^ in which case, the interfacial surface tension becomes: *γ*_*w*−*pcm*_ = *γ*_*pcm*(*a*)_ − *γ*_*w*(*a*)_*cosθ*. Inserting the values of the surface energy of liquid n-octadecane in air (*γ*_*pcm*(*a*)_), which is 21.6 mN/m, surface energy of water in air (*γ*_*w*(*a*)_), which is 72 mN/m, and the water droplet contact angle (θ = 82°), the interfacial energy (*γ*_*w* − *pcm*_) yields 11.58 mN/m. The force balance along the vertical direction remains critical to access the immersing (sinking) state of the droplet during the sliding on the inclined surface. The vertical force balance in relation to partial immersing of the droplet in the liquid n-octadecane film yields:

2 $${F}_{\gamma }\,\sin ({\theta }_{c}+\alpha )+{F}_{B}-W={m}_{d}{a}_{d}$$

where *θ*_*c*_ is the filling angle, which defines the position of the contact ring reference to the vertical axis (see S1), α is the contact angle of the water droplet cap at the ridge rim, *F*_*B*_ is the buoyancy force, *W* is the weight of the droplet, *m*_*d*_ is the droplet mass and a_d_ is the vertical acceleration of the droplet. The details of the formulation of surface tension force are given in Supplementary Material S1. It should be noted that the immersion takes place when the droplet weight (*W*) overcomes the sum of buoyancy and the vertical component of the surface tension force. After assuming that the geometric feature of the droplet cap can be simplified as the sphere cap, then, the vertical acceleration of the droplet $(\frac{{d}^{2}h}{d{t}^{2}})$ during immersion into the liquid n-octadecane film can be written as:

3 $$\frac{{d}^{2}h}{d{t}^{2}}=-\frac{1}{{\rho }_{w}C}\{{\rho }_{pcm}g[C-3\pi {h}^{2}({r}_{o}-h)]+2\pi \sqrt{(2{r}_{o}-h)h}{\gamma }_{w-pcm}\,\sin ({\theta }_{c}+\alpha )-{\rho }_{w}\,gC\}$$

where ${r}_{o}=\frac{4}{9}{h}_{o}(\frac{{r}_{o}^{3}}{{h}_{o}^{3}}+1)$ and $C=4\pi {h}_{o}[{h}_{o}^{2}(\frac{{r}_{o}^{3}}{{h}_{o}^{3}}+1)-1]$. Here, *ρ*_*w*_ is the density of water, *ρ*_*pcm*_ is the density of liquid n-octadecane, *h* is the spherical cap height (varying with immersion), *h*_*o*_ is the initial height of the spherical cap (prior to immersing), *r*_*o*_ is the initial diameter of the droplet spherical cap when it is first time deposited on the liquid n-octadecane surface. and *g* is the gravitational acceleration. It should be noted that for a fixed volume of droplet, *r*_*o*_, and *h*_*o*_ remain constant for known volume of droplet. Formulation of the droplet immersion $(\frac{{d}^{2}h}{d{t}^{2}})$ in the liquid n-octadecane is given in Supplementary Material S1. Eq. 3 is a non-linear second order differential equation, which can be solved numerically after introducing the initial conditions such as at *t* = 0, *h* = *h*_*o*_, and *dh*/*dt* = 0. The analytical solution of for the immersion velocity in Eq. 4 is complicated due to non-linear form of the equation. However, experimental data for the ridge height variation, due to the cloaking of n-octadecane on the water droplet, and for the depth of immersion of the droplet into the liquid n-octadecane, due to vertical force balance, can be compared along the inclined surface after considering the force balance in Eq. 3. The findings revealed that the depth of immersion predicted from Eq. 4 is in the order of ~38 μm on set of droplet formation on the film with inclination angle of 4°.

**Figure 5:**
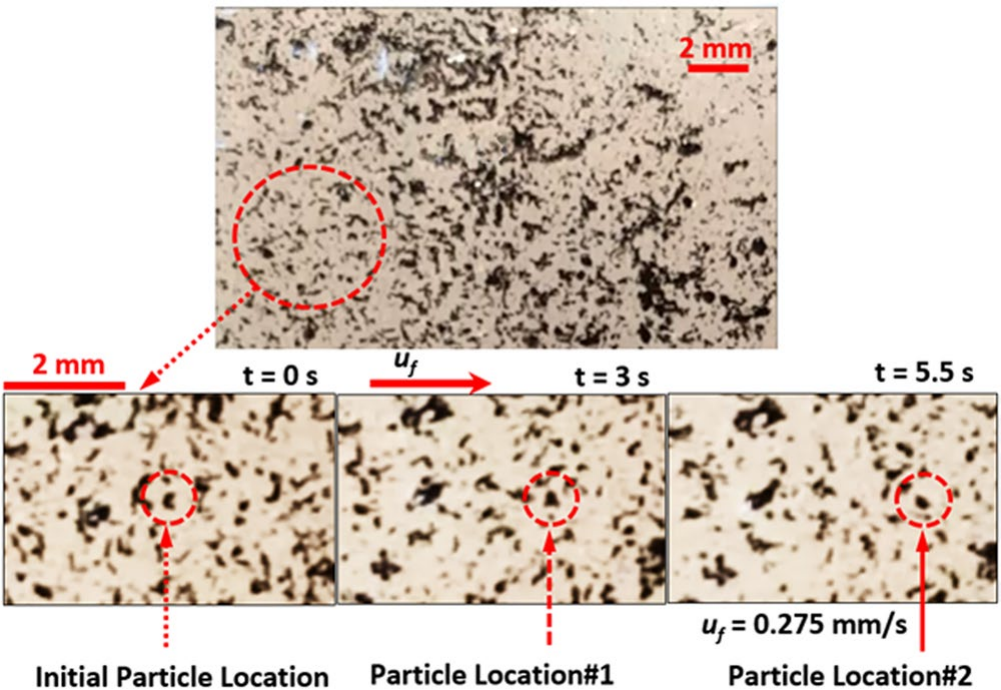
Images of tracking of carbon nanotube clusters in inclined thin film of liquid n-octadecane at various periods.

The droplet movement on the inclined liquid surface is associated with the force and energy balances in accordance with the moving droplet and the flowing thin liquid film of n-octadecane under the gravitational force due to the inclination. In order to assess the velocity of the liquid film on the textured surface, experiments are carried out. In this case, the solid n-octadecane particles are mixed with 1% (volume) of carbon nanotubes, and the mixture is liquefied and dip coating facility is used to coat the textured surface with the mixture. The n-octadecane coated film is liquefied on the sample surface and the sample is kept horizontally. Later, the n-octadecane coated sample is inclined and the inclined liquid film of n-octadecane flows on the sample surface under the gravity. The optical microscopic system integrated with a high-speed camera is used to monitor of the velocity of the clustered carbon nano-tubes in the inclined liquid film. Temperature of the textured surface and the liquid film is kept constant during the experiments. The tests are re-conducted eight times to secure the repeatability of the velocity data. Figure 5 shows the microscopic images of the carbon nano-tube clusters produced from the high-speed camera data and the fluid velocity at the surface of the inclined liquid n-cotadecane film.

In addition, the flow velocity in the inclined n-octadecane film can be formulated using the analogy of a flow falling film^34^. The velocity variation along the inclined surface was formulated previously incorporating the uniform film thickness analogy^34^. This results in ${u}_{f} \sim \frac{{\rho }_{pcm}\,g{h}_{t}^{2}cos\delta }{2{\mu }_{pcm}}$. Here, *ρ*_*pcm*_ is the density of the liquid n-octadecane, *g* is the gravitational acceleration, *h*_*t*_ is the liquid film thickness, *δ* is the inclination angle of the liquid film surface, and *μ*_*pcm*_ is the dynamic viscosity of the liquid film. The inclination angle (*δ*) is measured as the angle between the lateral direction along the surface and the horizontal line. The expression for *u*_*f*_ is valid for the region far away from the film beginning and ending, and it corresponds to the maximum velocity in the film, which occurs at the liquid film surface^35^. However, the expression for *u*_*f*_ does not consider the capillary effect in the film. In addition, the equation for the flow field in the micro-film on the inclined surface subjected to the gravity only is mainly governed by the stoke flow. In this case, the film thickness under the gravitational flow in the inclined film (*δ *≤ π/2, where *δ* is the inclination angle) is related to the capillary number ($Ca=\frac{{\mu }_{pcm}u}{{\gamma }_{pcm}}$, here *u* is the flow, *μ*_*pcm*_ is the fluid dynamic viscosity, *γ*_*pcm*_ is the surface tension)^36^. Therefore, the flow equation for inclined liquid film takes the form:

$$\frac{\partial {h}_{f}}{\partial t}=\nabla .[{h}^{3}\nabla {\nabla }^{2}{h}_{f}]-D\nabla .[{h}^{3}\nabla {h}_{f}]+\frac{\partial {h}_{f}^{3}}{\partial x}=0,$$

where *h*_*f*_ is the normalized film thickness ($\overline{{h}_{f}}/{h}_{c}$., here $\overline{{h}_{f}}$ is the film thickness, *h*_*c*_ is the film thickness far behind the film front), *x* is the distance along the inclined surface, and *D* is the dimensionless parameter (*D* = (3*Ca*)^1/3^*cotδ*, here *Ca* is the capillary number, *δ* is the inclination angle of the liquid film)^37^. The analytical solution for the flow equation in the film is complicated; however, incorporating the solution strategies sgested in the previous study^38^, the flow velocity can be obtained. In this case, the flow velocity at the film surface obtained from simplified form^35^, is in the order of 1.55 mm/s, experimental findings are in the order of 0.28 mm/s and partial differential equation^38^ results in the order of 0.21 mm/s. The experimental finding for surface velocity of the film agrees with that of the differential equation solution. Moreover, previously, the pinning (adhesion) force is formulated incorporating the advancing and receding angles of the droplet along the tree-phase contact line^39^; however, the droplet motion is governed by the sliding on a small angle of inclined surface and the correct assessment of droplet advancing and receding angles becomes difficult at the three-phase contact line. In addition, the surface of the liquid n-octadecane film moves under the gravitational influence, which in turn forces the droplet to display similar motion to the liquid film on the surface. Since the droplet velocity on the inclined liquid surface and the liquid film velocity under the droplet is not same, a resisting work has been done by the droplet against the pinning resistance for the condition of *V*_*d*_ > *u*_*f*_ (where *V*_*d*_ is the droplet translational velocity on the liquid film surface and *u*_*f*_ is the liquid film velocity on the surface). Hence, the work done against the pinning resistance of the droplet along the incremental lateral distance *ΔL* in the lateral direction on the inclined liquid n-octadecane surface (*W*_*p*_) is:

4 $${W}_{p}=2\pi {\gamma }_{w-pcm}rcos\,({\theta }_{c}+\alpha ){\rm{\Delta }}L$$

The droplet partially immerses in the liquid n-octadecane film, which in turn results in a shear resistance at the droplet bottom and at the rim of the ridge tip around the droplet. The shear forces can be formulated in terms of the rate of fluid strain at the droplet bottom and at the rim of the ridge tip. The liquid film velocity on the solid surface is same order of the slip velocity, which is related to the slip length. It should be noted that the slip length (*b*) at the liquid solid interface is formulated by $\frac{b}{L}=\frac{A}{{\varphi }^{2}}-B$, where *A* and B are the constant, *ϕ* is the solid fraction, and *L* is the length scale^40,41^. The solid fraction can be estimated for the droplet contact angles on the flat and textured surfaces after deposition of functionalized silica particles^42^. The solid fraction (*ϕ*) is related to the static contact angle for the textured (silica particles deposited surface) and the flat surfaces by *cosθ*_*tex*_ = [*ϕ*(*θ*_*flat*_ − 1)+ 1], where *θ*_*tex*_ is the droplet static contact angle on the textured surface and *θ*_*flat*_ is the droplet static contact angle on the flat surface^42^. For the texture surface, the contact angle is 160° and it is 75° on the flat surface; therefore, the solid fraction yields in the order of *ϕ* = 0.12. For a small solid fraction, the slip length (*b*) can be approximated by $b \sim \frac{{\mu }_{w}}{{\mu }_{pcm}}(1-\varphi ){h}_{t}$, where *μ*_*w*_ is the dynamic viscosity of water, *μ*_*pcm*_ dynamic viscosity of liquid n-octadecane, and *h*_*t*_ is the liquid film height (thickness). The dynamic viscosity of water is 0.7644 × 10^−3^ Pa.s at 305 K and dynamic viscosity of liquid n-octadene at the same temperature is 3.677 × 10^−3^ Pa.s^32^, and the oil film thickness is in the order of 50 μm; hence, the slip length becomes 19.65 μm. The slip velocity at the droplet bottom is related to the slip length in the form of ${u}_{s}=\frac{b}{\mu }{\tau }_{wi}$, where *τ*_*wi*_ is the shear stress at the interface^7^ and the the interfacial shear stress for the thin film can be simplified via couette flow analogy, i.e. ${u}_{s} \sim \frac{b}{{h}_{t}}{u}_{f}$. Since the droplet sliding velocity remains higher than the liquid n-octadecane surface velocity at the water droplet-n-octadecane interface, a shear stress is developed at the interface. The shear stress at the water droplet side is in the order of $\sim {\mu }_{w}\frac{(V-{u}_{f})}{{l}_{m}}$, where *μ*_*w*_ is the dynamic viscosity of water and *l*_*m*_ is the distance from the interface to the droplet center of mass^7^. The shear stress at the n-octadecane interface is in the order of $\sim {\mu }_{pcm}\frac{({u}_{f}-{u}_{s})}{{h}_{t}}$. Since the shear stresses at water droplet and n-octadecane sides are same, equating the shear stresses at the interface results in:

5 $$\frac{V}{{u}_{f}} \sim 1+\frac{{\mu }_{pcm}}{{\mu }_{w}}\frac{{l}_{m}}{{h}_{t}}(1-\frac{b}{{h}_{t}})$$

Since *b*/*h*_*t*_ is in the order of 0.39, which results in almost 60% less than the value presented in the previous study^7^. Nevertheless, the ratio of $\frac{{\mu }_{pcm}}{{\mu }_{w}}\frac{{l}_{m}}{{h}_{t}}\gg 1$ and it gives rise to *V* > *u*_*f*_, which agrees with the experimental observations. However, from the high-speed camera data, the droplet sliding is the only mechanism governing the dynamic motion of the droplet on the liquid n-octadecane film.

**Figure 6:**
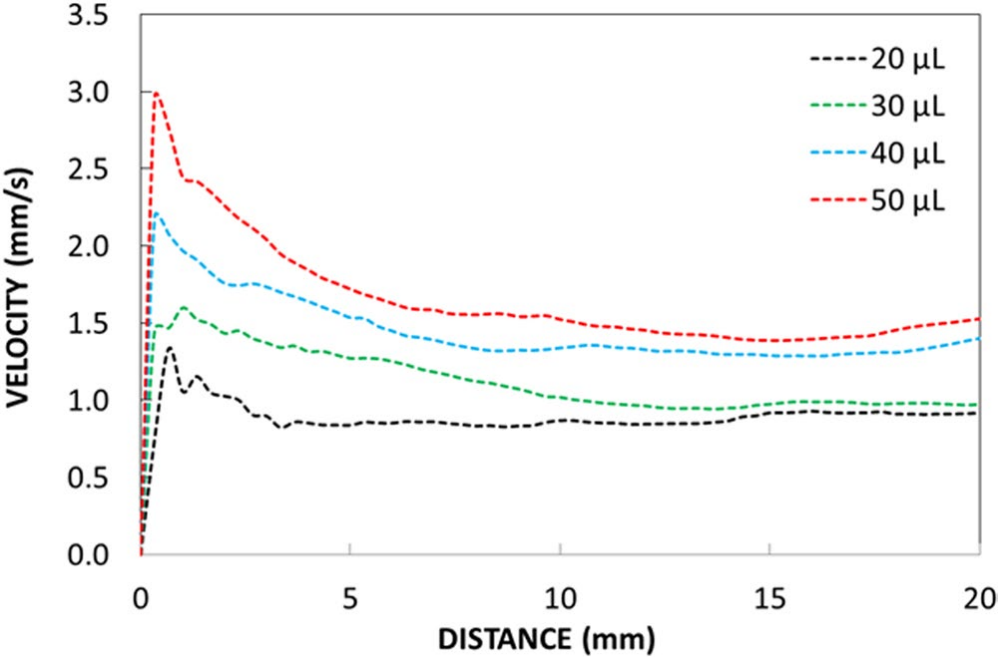
Droplet transition velocity on inclined thin film of liquid n-octadecane film with distance along the surface for various droplet volumes.

**Figure 7:**
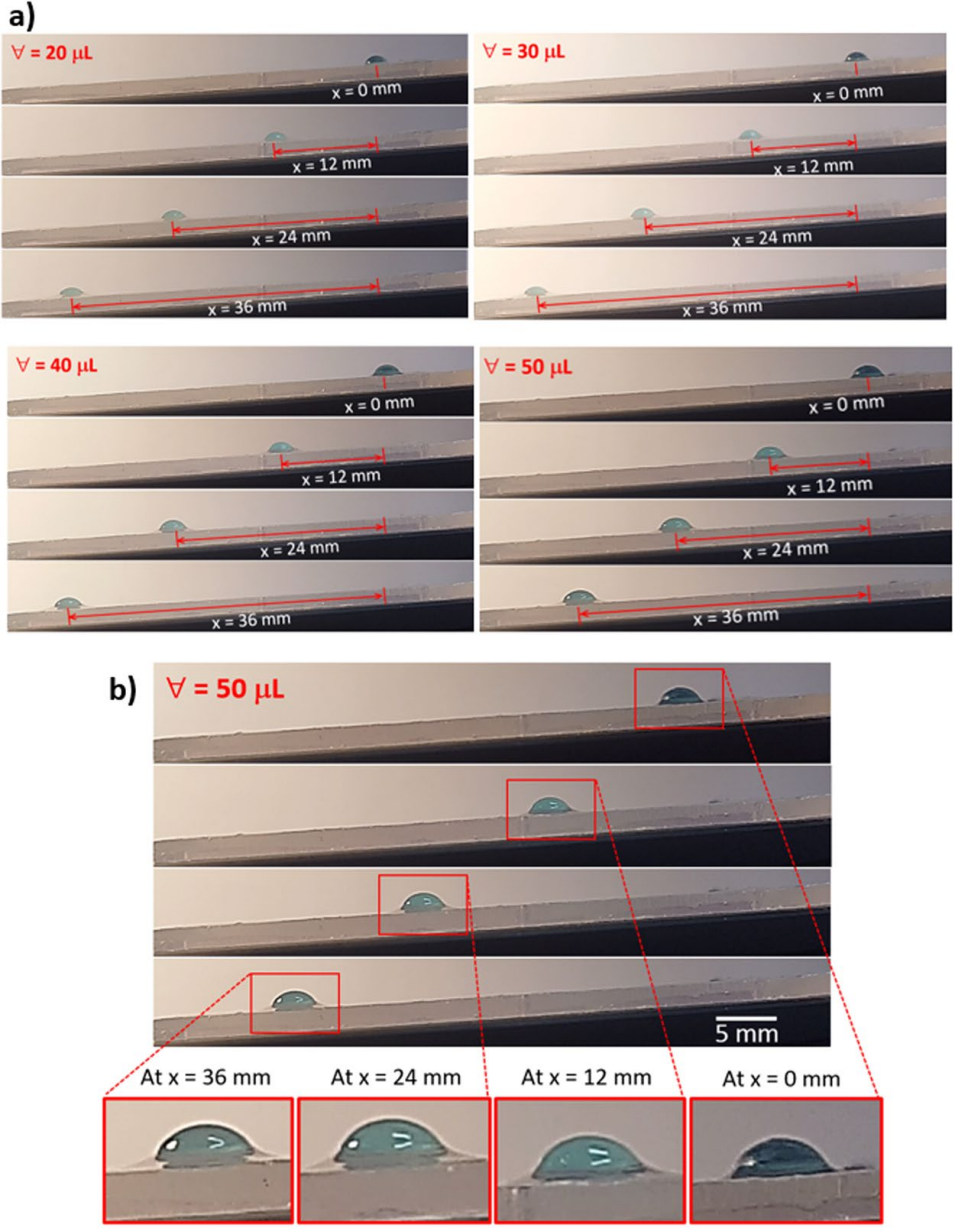
Images of droplet on inclined thin film of liquid n-octadecane film for various droplet volumes: (**a**) side view of droplet at different locations on inclined surface, and (**b**) n-octadecane cloaking of water droplet during droplet transition on inclined surface.

On the other hand, energy dissipation takes place during droplet movement on the inclined surface, which can be related to fluid friction across the interface of water droplet-liquid n-octadecane surface, work done against drag because of air and partial immersion of the droplet in a liquid n-octadecane film, work done against pinning of droplet due to lateral surface tension force, and work done against resistance of ridges around the droplet. The viscous dissipation, due to shear resistance associated with the droplet volume, can be formulated in line with the previous work^43^, which yields: $\sim {\mu }_{w}\frac{V}{{l}_{m}}\pi {r}_{o}^{2}$, where *r*_*o*_ is the base radius of the droplet. The geometric relations between the base radius (*r*_*o*_) and height of center of mass (*l*_*m*_) can be written as $\frac{{r}_{o}}{{l}_{m}}=\frac{4}{3}\frac{sin\theta (2+cos\theta )}{{(1+cos\theta )}^{2}}$ ^7^. The energy dissipated due to interfacial shear resistance of the droplet and the liquid film on the surface can be formulated after incorporating the method adopted in the early study as $\sim {\mu }_{w}(\frac{V-{u}_{f}}{{l}_{m}})\pi {r}_{o}^{2}$ ^7^. The work done due to viscous dissipation in the wetting ridge can be approximated as $\sim {\mu }_{pcm}(\frac{V-{u}_{f}}{{l}_{ridge}})2\pi {r}_{o}{l}_{ridge}$, where *l*_*ridge*_ is the ridge height around the droplet meniscus. This can be simplified as $\sim {\mu }_{pcm}(V-{u}_{f})2\pi {r}_{o}$ ^7^. Since the water droplet partially immerses in the film because of the vertical force balance (Eq. 2), the water volume in the film suffers from the drag force during the droplet motion. In addition, the droplet volume outside of n-octadecane undergoes an air drag. The work done due to the drag in air and in liquid n-octadecane contributes to the energy dissipation during the droplet motion on the inclined liquid n-octadecane film. Since the small volume of the droplet is immersed in n-octadecane, and the liquid film thickness is small, the drag force can be approximated by Faxen’s law^44^, in this case, the drag force exerted on the droplet in the liquid film onset of the droplet motion can be written as ~6*πμ*_*pcm*_*h*_*pcm*_(*V* − *u*_*f*_) where *h*_*pcm*_ represents the droplet height in the liquid film. Here, the droplet height immersed in the film (*h*_*pcm*_) can be determined from Eq. 3, which is in the order of ∼38 μm for droplet volume of 50 μL, which is close to the liquid film thickness (40 μm). In addition, the air drag can be approximated by ~*C*_*d*_*ρ*_*a*_*A*_*c*_*V*^2^, where *C*_*d*_ is the drag coefficient (which is in the order of ∼0.2–0.5), *ρ*_*a*_ is the air density, *A*_*c*_ the cross-sectional area of droplet floating above the liquid n-octadecane film, which can be determined by use of Eq. 3, i.e. after considering the droplet height above the surface of n-octadecane (*h*_*o*_ − *h*_*pcm*_, where *h*_*o*_ is total droplet height in the vertical direction), *A*_*c*_ can be determined. *A*_*c*_ varies with the droplet size within the order of 1.2 × 10^−6^ m^2^ to 2.6 × 10^−6^ m^2^. The conservation of energy yields the formulation of droplet translational velocity on the liquid film surface. Hence, the change of the droplet potential energy remains same as the sum of the energy dissipation and kinetic energy change during the droplet transition on the inclined film of the liquid n-octadecane surface. It, therefore, satisfies the equation *ΔE*_*Tot*_ = *ΔE*_*loss*_ + *ΔE*_*kinetic*_, where *ΔE*_*tot*_ (*ΔE*_*tot*_ = *m*_*d*_*gΔh*, where *m*_*d*_ is the mass of the droplet, *g* is the gravitational acceleration, and *Δh* is the elevation between the droplet location and the reference level and it represents the change of gravitational potential energy of the droplet along the inclined hydrophobic surface. *ΔE*_*loss*_ is the dissipated energy, which can be written in the form of *ΔE*_*loss*_ = *ΔE*_*pinning*_ + *ΔE*_*shear*_ + *ΔE*_*drag*_, where *ΔE*_*pinning*_ is the work done against droplet pinning resistance, and *ΔE*_*shear*_ is the total viscous dissipation due to shear, *ΔE*_*drag*_ is the work done against the liquid film and the air drags during the movement of the droplet. The energy dissipation due to pinning of the droplet is given in Eq. 4. The the total viscous energy dissipation due to fluid friction and drag forces along the incremental distance *ΔL* on the surface is in the order of ∼*ΔL*(${\mu }_{w}\frac{V}{{l}_{m}}\pi {{r}_{o}}^{2}+{\mu }_{w}(\frac{V-{u}_{f}}{{l}_{m}})\pi {{r}_{o}}^{2}+{\mu }_{pcm}(V-{u}_{f})2\pi {r}_{o}$ + $6\pi {\mu }_{pcm}{h}_{pcm}(V-{u}_{f})$ + $\,{C}_{d}{\rho }_{a}{A}_{c}{V}^{2}$) and the work done against droplet pinning is in the order of $2\pi {\gamma }_{w-pcm}Rcos\,({\theta }_{c}+\alpha )\Delta L$. The kinetic energy of the droplet at the any location on the inclined surface is in the order of $\frac{1}{2}{m}_{d}{(V-{u}_{f})}^{2}$. The droplet mass (*m*_*d*_) can be written as $\,{m}_{d} \sim {\rho }_{w}3\pi {h}_{o}^{2}({r}_{o}-{h}_{o})$ after consideration of the droplet shape resembling the spherical cap. Here, *h*_*o*_ is the maximum droplet height in the vertical direction, *r*_*o*_ is the droplet radius at the droplet meniscus. The gravitational potential energy change of the droplet along the incremental distance *ΔL* on the surface is in the order of $\sim \,{\rho }_{w}3\pi {h}_{o}^{2}({r}_{o}-{h}_{o})gsin\delta$, where *δ* is the inclination angle of the liquid n-octadecane film surface. The details of the formulation of droplet velocity on the inclined liquid n-octadecane film are given in Supplementary Material S1. In order to assess the influence of viscous energy dissipation and the work done against the drag and pinning forces on the droplet kinetic energy, the scale analysis is carried out for each energy dissipation term in the energy balance equation for droplet motion on the liquid n-octadecane film (Supplementary Material S1) In the scale analysis, the gravitational potential energy change along the distance *ΔL* is considered as the reference towards normalizing all the dissipation terms in the energy equation (Supplementary Material S1). Hence, the ratio of the viscous energy dissipation over the gravitational potential energy change is in the order of 0.03815 and the ratio of the work done against the drag forces over the gravitational potential energy change is in the order of 0.0086. The ratio of the work done against the pinning force over the gravitational potential energy change is in the order of 0.0875. Hence, the work done against the droplet pinning (retention) is the largest among the energy dissipation, due to friction and drag forces during, the translational motion of the droplet on the inclined liquid film of n-ocdecane. Figure 6 shows translational velocity of the droplet obtained from the experiment incorporating the high-speed camera data on the inclined liquid film of n-octadecane surface for various droplet sizes. It should be noted that the high-speed camera recording was repeated by ten times and the images recorded for the water droplet at various locations are compared in terms of the droplet wetting length on the surface, the droplet width, and the droplet height. The measurement errors are estimated as the followings: (i) it is in the order of 4% in terms of variations of droplet height, droplet wetting length, and droplet mid-width, and (ii) it is in the order of 3% in terms variations of the dynamic droplet contact angle. It is observed from the high-speed camera data that the droplet slides rather than rolls on the liquid n-octadecane surface. This is related to the retention force due to pinning and interfacial resistance of the droplet and the liquid film surface. The droplet translational velocity increases rapidly and it reaches almost the terminal velocity after the short distance from the starting point on the inclined hydrophobic surface, which is true for all droplet volumes incorporated in the present study. Initial acceleration of the droplet is associated with the force balance between the gravitational force and retarding forces due to pinning, interfacial shear, and drag. This indicates that gravitational force remains significantly higher than those of the retarding forces. The translational velocity predicted from the energy balance around the droplet (Supplementary Material S1, Eq. 20) is within the order of ∼2 mm/s for 50 μL droplet volume; however, depending of the droplet radius (*r*_*o*_) and droplet height (*h*_*o*_) on the three-line contact line the droplet velocity changes; in which case, decreasing *r*_*o*_ and *h*_*o*_ results in smaller translational velocity. The findings of Eq. 20A agree well with the experimental data for the terminal velocity of the droplet, which is within the order of 1.85 mm/s for 50 μL droplet volume. The attainment of large translational velocity for large volume droplets is attributed to the gravitational force acting on the droplet, which increases with increasing droplet mass. Figure 7a shows the optical images on the droplet on the liquid n-octadecane surface for various of distances on the inclined thin film of liquid n-octadecane and various droplet sizes while Fig. 7b shows the ridges formed around the droplet during droplet transition along inclined thin film of liquid n-octadecane. The distance taken by the droplet increases slightly by increasing droplet volume (Fig. 7a), however, this difference is small, which can be observed from Fig. 6, i.e. the droplet terminal velocities are close to each other. The ridges are formed around the droplet (Fig. 7b) and the ridge size remains almost same as the droplet reaches its terminal velocity.

## Conclusion

The exchange of hydrophobic to hydrophilic surface states is investigated via introducing a thin film of phase change material on the textured surface. The surface of the polycarbonate wafers is solution crystallized to achieve hierarchal distribution of micro/nano pillars consisting of fibrils and spherules. Acetone crystallization results in hydrophobic surface with the average contact of 130° and the corresponding hysteresis of about 36°. To lower the contact angle hysteresis and improve the contact angle, the crystallized polycarbonate surface is coated with the nano-size silica particles. The deposition provides the average contact angle of 158° and the corresponding hysteresis of about 2°. To alter reversibly the wetting state on the surface, a film of n-octadecane coating is applied on the textured surface. The coating thickness of the liquid film is determined from the ellipsometer data and thickness is found to be about 40 μm. In this case, when n-octadecane solidifies surface becomes hydrophobic with the average contact angle of 140° and the hysteresis about 8°. However, when the coating liquefies, the average contact angle becomes 82°. The surface velocity of the liquid film is measured and it is in the order of 0.28 mm/s. The droplet behavior on the inclined liquid film surface is examined and equations for force balance is developed incorporating the gravitational acceleration and retention forces including pinning, interfacial shear, and drag forces. A mathematical relation is presented for the droplet translational velocity incorporating the work done due to gravitational and retarding forces. The contribution of the pinning force to the overall retarding force is the highest as compared to those interfacial viscous and drag forces. The translational velocity, which is obtained from the high-speed camera, increases sharply along the short length of the liquid n-octadecane surface from its initiation. The droplet reaches almost its terminal velocity as the distance from the location of the droplet initiation increases along the inclined liquid n-octadecane surface. Increasing the droplet volume enhances the droplet translational velocity because of the increased gravitational force with increasing with droplet mass. For the first time, the reversible exchange of the wetting state of the hydrophobic surface is provided through introducing a thin layer of the phase change material (n-octadecane). It also discusses the droplet mobility on the inclined liquid n-octadecane surface and provides useful information about the droplet pinning and sliding when the liquid film has hydrophilic characteristic.

## Electronic supplementary material

Supplementary Information
