# Supplementary material for: Mobility of A Water Droplet on Liquid Phase of N-Octadecane Coated Hydrophobic Surface
Source: Sci Rep. 2018 Oct 10;8:15060. doi: 10.1038/s41598-018-33384-0 (PMC6180037; doi:10.1038/s41598-018-33384-0)
Supplement: Supplementary file 1 — Supplementary Information [file 41598_2018_33384_MOESM1_ESM.docx]

Supplementary Information

**MOBILITY OF A WATER DROPLET ON LIQUID PHASE OF N-OCTADECANE COATED HYDROPHOBIC SURFACE**

Bekir Sami Yilbas*^,1,2^, Haider Ali^1^, Abdullah Al-Sharafi^1^, Nasser Al-Aqeeli^1^ Numan Abu-Dheir^1^, Kahraman Demir^1^

^1^ Mechanical Engineering Department and Centre of Excellence in Renewable Energy, King Fahd University of Petroleum & Minerals, Dhahran, Saudi Arabia,

^2^ Center of Research Excellence in Renewable Energy (CoRE-RE), King Fahd University of Petroleum and Minerals (KFUPM), Dhahran 31261, Saudi Arabia

*Corresponding author. Email: [bsyilbas@kfupm.edu.sa](mailto:bsyilbas@kfupm.edu.sa); Phone: +966 3 860 4481

Consider n-octadecane film and immersion of a water droplet on the film surface. After assuming the semi-spherical geometry of the water droplet on the liquid phase, the vertical force balance gives rise to the droplet immersion in the liquid film. In line with Fig. S1, in which the droplet geometry - resembling a semi-spherical cap - and force diagram is shown schematically, the geometric features of the droplet can be formulated. The droplet radius on the meniscus of the three-phase contact line is:

$r=\sqrt{h\left(2r_{o}-h\right)}$ (1)

where *h* is the droplet height, *r_o_* is the radius of the spherical cap on the liquid n-octadecane surface.


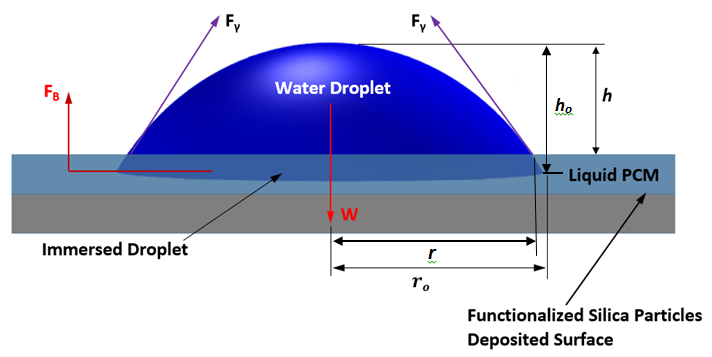


**Figure S1.** A schematic view of water droplet formed on the liquid phase of n-octadecane film when located horizontally and the force diagrams.

However, the spherical droplet volume after spreading on the liquid n-octadecane surface becomes a semi-spherical cap. The initial volume of the droplet occupying a semi-spherical cap yields:

$\forall=\frac{\pi}{3}h^{2}\left(3r_{o}-h_{o}\right)$ (2)

where $r_{o}=\sqrt{h_{o}\left(2R_{o}-h_{o}\right)}$ and *h_o_* is the maximum droplet height, which corresponds to spherical cap onset of its formation on the liquid n-octadecane surface.

However, droplet immerses into the liquid droplet depending on the force balance. In this case, the droplet volume remaining above the liquid surface of n-octadecane becomes:

$\forall=\frac{\pi}{3}h^{2}\left(3r-h\right)$ (3)

The volume displaced of liquid n-octadecane is:

(4)

or

$\forall_{disp}=\frac{\pi}{3}\left[h_{o}^{2}\left(3r_{o}-h_{o}\right)-h^{2}\left(3r_{o}-h\right)\right]$ (5)

The length of the 3-phase contact line is:

(6)

Consider Fig. S2, the vertical force balance for immersing water droplet occupying a semi-spherical cap yields:

$\sum F_{y}= F_{B}+F_{\gamma}-W=m_{d}a_{d}$ (7)

The buoyancy force is:

$F_{B}=\rho_{pcm}g\forall_{disp}=\frac{\pi}{3}\rho_{pcm}g\left[h_{o}^{2}\left(3r_{o}-h_{o}\right)-h^{2}\left(3r_{o}-h\right)\right]$ (8)

The surface tension force is:

(9)

The vertical component of the surface tension force yields:

$F_{v-\gamma}=F_{\gamma}sin(\theta_{c}+\alpha)$ (10)

where *θ_c_* is the filling angle, which defines the position of the contact ring reference to the vertical axis (Fig. S2), α is the contact angle of the water droplet cap at the ridge rim.

The droplet weight is:

$W=\rho_{w}g\forall_{in}=\frac{\pi}{3}\rho_{w}gh_{o}^{2}\left(3r_{o}-h_{o}\right)$ (11)

Inserting the forces into the vertical force balance equation, it yields:

$\begin{matrix} \frac{\pi}{3}\rho_{w}h_{o}^{2}\left(3r_{o}-h_{o}\right)a_{d}=\frac{\pi}{3}\rho_{pcm}g\left[h_{o}^{2}\left(3r_{o}-h_{o}\right)-h^{2}\left(3r_{o}-h\right)\right] \\ +2\pi\gamma_{w-pcm}\sqrt{h\left(2r_{o}-h\right)}\sin(\theta_{c}+\alpha)-\frac{\pi}{3}\rho_{w}gh_{o}^{2}\left(3r_{o}-h_{o}\right) \end{matrix}$ (12)


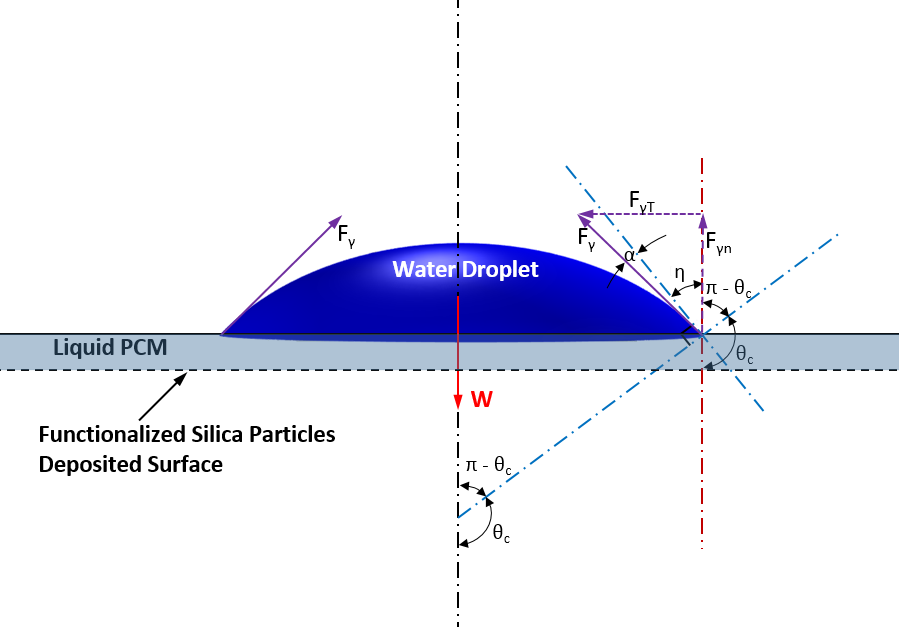


**Figure S2.** A schematic view of water droplet on liquid phase of n-octadecane when located horizontally and surface tension force diagram.

Since the term $\frac{\pi}{3}h_{o}^{2}\left(3r_{o}-h_{o}\right)$ is constant for a fixed droplet volume, i.e. $C=\frac{\pi}{3}h_{o}^{2}\left(3r_{o}-h_{o}\right)$, the droplet acceleration towards immersion into the liquid n-octadecane becomes:

$a_{d}=\frac{1}{\rho_{w}C}\left[\rho_{pcm}g\left(C-h^{2}\left(3r_{o}-h\right)\right)\right]+2\pi\gamma_{w-pcm}\sqrt{h\left(2r_{o}-h\right)}\sin(\theta_{c}+\alpha)-\rho_{w}gC$ (13)

or

$a_{d}=\frac{\rho_{pcm}}{\rho_{w}}g\left[1-\frac{h^{2}\left(3r_{o}-h\right)}{C}\right]+\frac{2\pi\gamma_{w-pcm}\sin(\theta_{c}+\alpha)}{\rho_{w}}\left[\frac{\sqrt{h\left(2r_{o}-h\right)}}{C}\right]-g$ (14)

In another form, the droplet vertical acceleration during immersion can be written as:

$\frac{d^{2}h}{dt^{2}g}=\frac{a_{d}}{g}=\frac{\rho_{pcm}}{\rho_{w}}\left[1-\frac{h^{2}\left(3r_{o}-h\right)}{C}\right]+\frac{2\pi\gamma_{w-pcm}\sin(\theta_{c}+\alpha)}{\rho_{w}g}\left[\frac{\sqrt{h\left(2r_{o}-h\right)}}{C}\right]-1$ (15)

After considering the water droplet volume is spherical prior to spreading on the liquid n-octadecane surface and equating the volume of spherical droplet with that of semi-spherical cap, it results in:

$\frac{4\pi}{3}r_{d}^{3}=\frac{\pi}{3}h_{o}^{2}\left(3r_{o}-h_{o}\right)$ (16)

where *r_d_* represents the spherical droplet radius. Rearrangement results in:

$r_{o}=\frac{h_{o}}{3}\left(\frac{4r_{d}^{3}}{h_{o}^{3}}+1\right)$ (17)

Here, Eq. 17 can be used to replace ro in Eq. 15; in which case, acceleration of the droplet can be formulated in terms of the droplet radius when the droplet is spherical prior to spreading and forming a spherical cap. Eq. 15 is a nonlinear second order ordinary differential equation, which can be solved numerically.

The energy balance for the droplet over the incremental distance *ΔL* on the inclined liquid n-octadecane surface yields:

$$\rho_{w}3\pi h_{o}^{2}\left( r_{o}-h_{o} \right)\frac{V^{2}}{2}\sim$$

$\Delta L\left[ \rho_{w}3\pi h_{o}^{2}(r_{o}-h_{o})gsin\delta-\left( \begin{aligned} \mu_{w}\frac{V}{l_{m}}\pi{r_{o}}^{2}+\mu_{w}(\frac{V-u_{f}}{l_{m}})\pi{r_{o}}^{2}+\mu_{pcm}\left( V-u_{f} \right)2\pi r_{o} \\ +6\pi\mu_{pcm}h_{pcm}\left( V-u_{f} \right)+ C_{d}\rho_{a}A_{c}V^{2} \\ +2\pi\gamma_{w-pcm}r_{o}cos (\theta_{c}+\alpha) \end{aligned} \right) \right]$ (18)

Eq. 18 can be rearranged as:

$AV^{2}+BV+C=0$ (19)

where the coefficients are: $A=\frac{1}{2g\Delta Lp}-f$; $B=2a+\mu_{pcm}+d$; and $C=b+c+e+k$. Here, the variables are; $a=\frac{{\pi\mu}_{w}{r_{o}}^{2}}{l_{m}}$; $b={\frac{{\pi\mu}_{w}{r_{o}}^{2}}{l_{m}}u}_{f}$; ${c=2\pi\mu}_{pcm}r_{o}u_{f}$; $d=6\pi\mu_{pcm}h_{pcm}$; ${e=6\pi\mu_{pcm}h_{pcm}u}_{f}$; ${f=C}_{d}\rho_{a}A_{c}$; $k=2\pi\gamma_{w-pcm}r_{o}cos (\theta_{c}+\alpha)$; $m=2gsin\delta$; and $p=\frac{g}{3\pi\rho_{w}h_{o}^{2}(r_{o}-h_{o})}$.

Eq. 19 yields the relation for the droplet velocity *V*, i.e.:

$V\sim\frac{-(2a+\mu_{pcm}+d)+\sqrt{{(2a+\mu_{pcm}+d)}^{2}-4(\frac{1}{2g\Delta Lp}-f)(b+c+e+k)}}{2(\frac{1}{2g\Delta Lp}-f)}$ (20)

Eq. 20 gives the relation between the droplet velocity and the physical parameters affecting the energy dissipation due to the resisting forces.
